# Effects of various liquid-to-powder ratios on the compressive strength of calcium enriched mixture: Original research

**DOI:** 10.34172/joddd.2021.022

**Published:** 2021-05-05

**Authors:** Mohammad Forough Reyhani, Sheida Hosseinian Ahangarnezhad, Negin Ghasemi, Amin Salem Milani

**Affiliations:** ^1^Department of Endodontics, Dental and Periodontal Research Center, Faculty of Dentistry, Tabriz University of Medical Sciences, Tabriz, Iran; ^2^Dentist, Private Practitioner, Tabriz, Iran

**Keywords:** CEM cement, Compressive strength, Liquid-to-powder ratio

## Abstract

**Background.** Calcium-enriched mixture (CEM) cement has been introduced and marketed as a biomaterial for use in furcal perforation repair and apexogenesis procedures, in which the compressive strength that indicates the material’s resistance against crushing is of utmost importance. This study evaluated the effect of various liquid-to-powder ratios on CEM cement’s compressive strength.

**Methods.** One gram of the cement was mixed with 0.5, 0.34, and 0.25 mL of demineralized water and transferred to stainless steel molds (6 and 4 mm in height and diameter, respectively). Five cells in the mold were considered for each group. The compressive strength test was conducted using the universal testing machine after incubating for seven days under 95% humidity at 37°C. One-way ANOVA was applied for data analysis at *P* ≤ 0.05 significance level.

**Results.** The mean compressive strength in the liquid-to-powder ratios of 0.5, 0.34, and 0.25 were 3.4456, 3.2960, and 3.3485, respectively, with no significant differences between them.

**Conclusion.** Under this study’s limitations, changing the liquid-to-powder ratio did not affect CEM cement’s compressive strength.

## Introduction

Calcium-enriched mixture (CEM) cement is an endodontic biomaterial containing various compounds, including calcium oxide, calcium carbonate, calcium hydroxide, and calcium chloride.^1,2^ CEM cement has extensive applications in endodontics, including perforation repair, apexogenesis, and apexification; it is also used as a retrofilling material in surgery due to its favorable properties, e.g., setting in the presence of blood and humidity, anti-bacterial activity, proper sealing, and biocompatibility.^3^ The clinical application of this material is similar to mineral trioxide aggregate (MTA).^4^ CEM cement has no MTA disadvantages like long setting time and difficult handling.^5-7^

Physical properties of endodontic biomaterials, such as compressive strength, are essential in conditions in which these materials are exposed to occlusal forces.^8^ Some of the clinical conditions similar to this include this material’s application in the pulp capping, apexogenesis, and furcal perforation repair procedures, in which the material is under the load of the repair material and occlusal forces; therefore, it should resist these loads and not collapse.^8,9^

The compressive strength is an indicator of the hydration reaction of powder particles during setting and is also a reflection of the setting method of the material.^10^

Studies have demonstrated that factors like environment pH, mixing methods, and contamination with blood affect CEM cement’s compressive strength.^9,11,12^

CEM cement’s compressive strength improves in the acidic and alkaline environments; however, an alkaline environment results in better results.^12^ In various mixing methods, the maximum CEM cement compressive strength is achieved with manual mixing.^11^ Blood has no adverse effects on this material’s compressive strength; however, blood contamination of the cement makes it brittle.^9^

Like MTA, this biomaterial is a hydrophilic cement, and it is estimated that its physical properties can be affected by different liquid-to-powder ratios.^13,14^ An investigation into the effect of various liquid-to-powder ratios of MTA showed that an increase in the amount of liquid increased radiopacity, an increase in the released calcium ions, and an increase in the environmental pH, with no significant effect on the setting time.^15^

A study on the effect of various liquid-to-powder ratios on CEM cement showed that, unlike MTA, the material’s push-out strength decreased along with an increase in the liquid-to-powder ratio.^14^

The manufacturer has not proposed any exact mixing ratio for CEM cement, and in most cases, the operator’s intended stability is considered in mixing.^15^ This study investigated the effect of different liquid-to-powder ratios on the compressive strength of this cement. The proportions of 1:2, 1:3, and 1:4 were considered to compare this study’s results with similar previous studies.

## Methods

The compressive strength was measured according to the ISO 6876:2012 standard.^16^ Therefore, there were three groups, as follows, in terms of the liquid-to-powder ratios:

1. CEM cement with 1:2 liquid-to-powder ratio (1 g of powder with 0.5 mL of liquid)
2. CEM cement with 1:3 liquid-to-powder ratio (1 g of powder with 0.34 mL of liquid)
3. CEM cement with 1:4 liquid-to-powder ratio (1 g of powder with 0.25 mL of liquid)

**Figure 1:**
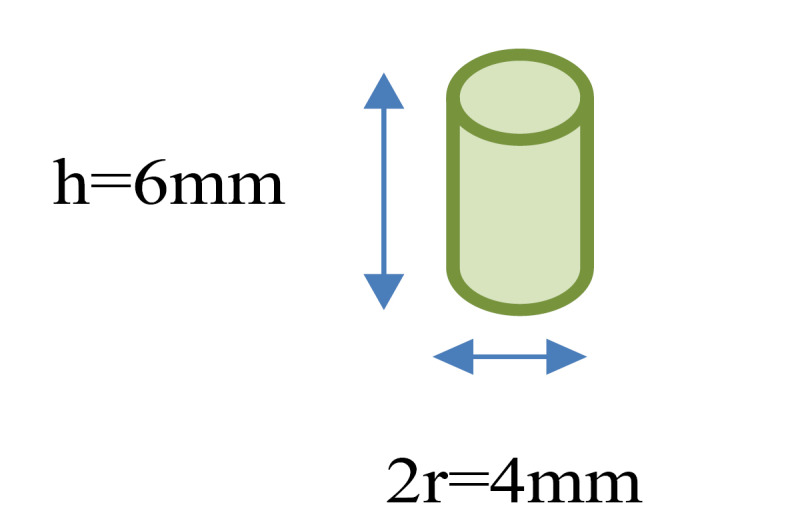
Sample size

One gram of CEM cement, measured by the digital weighing machine, was mixed with 0.5, 0.34, and 0.25 mL of demineralized water. Each group’s liquid volume was measured with a graduated pipette at 1:0.01 mL and then added to the powder with a 1-mL diabetes syringe. The mixture was then transferred to stainless steel molds (6 mm in height and 4 mm in diameter) (Figure 1).

Five cells in the mold were considered for each group. First, the materials and the instruments were incubated at 23±1°C for an hour before use. Next, CEM cement was gradually placed in stainless steel molds and packed by a proper condenser with medium force. Before CEM cement positioning, the inner surface of the molds was coated with paraffin.

The extra material was removed using a cotton roll. Therefore, a wet piece of gauze was positioned in the mold’s upper section to simulate the clinical condition. The samples were incubated for seven days at 37°C and 95% humidity. The samples were retrieved from the molds after seven days and examined for the absence of bubbles and cut edges.

The accepted samples underwent a compressive strength test. Hounsfield universal testing machine (Hounsfield Test Equipment, Model HSK-S, Salfords, Redhill, Surrey, England) was used to this end.

The crosshead speed was set at about 1 mm/min. The prepared blocks were positioned one by one in a longitudinal direction to perform the tests. The maximum load to break the samples was recorded in Newton (N).

The compressive strength was then calculated in MPa, with the following formula:

CS=4F/(πd^2^)

F = maximum applied load (N)

d = the cylindrical specimen diameter (mm)

SPSS 16 (SPSS Inc., Chicago, IL, USA) was used for data analysis. The Kolmogorov–Smirnov test was applied to evaluate data normality. One-way ANOVA was used to compare the results at *P* ≤ 0.05 significance level.

## Results

Table 1 presents the means and standard deviations of compressive strengths. The Kolmogorov–Smirnov test was applied to assess the normal distribution of data. The results of this test are presented in Table 2. Based on the results, the data distribution was normal under all three conditions.

**Table 1:** Means ± SD of compressive strengths in study groups

**Liquid-to-powder ratio**	**Mean**	**SD**
0.5	3.4456	0.4556
0.34	3.2960	0.2099
0.25	3.3485	0.1684

**Table 2:** Normal distribution was tested using the Kolmogorov-Smirnov test

		**1:2**	**1:3**	**1:4**
N		5	5	5
Normal parameters	Mean	3.445660	3.296060	3.348580
	SD	0.4556340	0.2099729	0.1684015
Most extreme differences	Absolute	0.355	0.278	0.197
	Positive	0.355	0.278	0.158
	Negative	-0.191	-0.160	-0.197
Test statistic		0.355	0.278	0.197
Asymp. Sig. (2-tailed)		0.069	0.200	0.200

The results of the three different modes were compared using one-way ANOVA. The results are presented in Table 3. The compressive strengths were not significantly different between the three study groups.

**Table 3:** Comparison of compressive strength in the study groups

	**Sum of squares**	***df***	**Mean square**	**F**	***P*** ** value**
Between groups	0.058	2	0.029	0.309	0.740
Within groups	1.120	12	0.093		
Total	1.178	14			

## Discussion

This study evaluated the effects of various liquid-to-powder ratios on CEM cement’s compressive strength. This study’s results demonstrated that the liquid-to-powder ratio did not significantly affect CEM cement’s compressive strength.

Compressive strength is an essential characteristic of endodontic biomaterials, influenced by different factors, such as mixing method, condensation force, and blood presence in the environment.^9,17^ These physical properties are important because the use of this cement in the pulp cap, apexogenesis, and furcal perforation repair procedures requires sufficient compressive strength against the functional loads and forces resulting from placing the restorative materials on it.^8,10^

The experimental procedure of this study was performed in terms of the ISO 6876:2012 standard.^16^ The temperature and humidity for all the three groups of samples were similar. Placing and condensation were performed by one operator and with a specific condenser size to apply the same amount of load on the samples during packaging. The mixing technique was manual in all the samples.

Similar to other hygroscopic cements, the liquid-to-powder ratio can affect the cement properties. Previous studies indicated that a high liquid-to-powder ratio decreased the compressive and push-out bond strength of MTA.^18^

Also, it has been reported that an increase in the liquid-to-powder ratio increased the solubility and porosity of MTA.^19^

No specific liquid-to-powder ratio has been proposed for the CEM cement mixing by the manufacturer or previous studies; therefore, various ratios have been used in previous studies.^11,20^

Liquid-to-powder ratios were set at 1:2, 1:3, and 1:4. The reason for selecting these ratios was to compare the results with those of similar previous studies. Various methods have been used to prepare cylindrical samples of the cement for a compression test. According to ISO 9917-1:2007 standard,^21^ a two-piece mold pattern, not affected by cement, is recommended. In this study, a two-piece stainless-steel mold was used. An advantage of these two-piece molds is that the samples can easily be removed from the molds by a gentle force. After placing the materials, the molds were wrapped in a piece of wet sterilized gauze to simulate clinical conditions. Of course, one of the limitations of the present study was the absence of PBS. The material was exposed to the tissue fluid from one side in the clinical condition, which contained phosphate-buffered saline. Phosphate participates in the setting of biomaterials, which was observed in CEM cement.^22^

According to this study, variations in the liquid-to-powder ratio did not significantly affect the CEM cement compressive strength, which is different from the results of a study by Shojaee et al. In this study, an increase in the liquid-to-powder ratio decreased the CEM cement compressive strength.^23^

In the present study, the compressive strength was higher than that in the study above, which can be attributed to the longer incubation time. In this study, the samples were incubated for seven days, which was longer than the research by Shojaee et al, in which they were incubated for about four days.

## Conclusion

According to this in vitro study, different liquid-to-powder ratios did not significantly affect CEM cement’s compressive strength.

## Authors’ contributions

MFR and NG planned the study. NG performed the literature review. SHA performed the experiments, drafted the manuscript, performed the experimental procedure, carried out statistical analyses, and interpreted the data. ASM critically revised the manuscript for intellectual content. All the authors have read and approved the final manuscript.

## Acknowledgments

The authors thank all the friends and staff members in the Oral and Medicine Department, who helped us complete the study.

## Funding

This study was supported by Vice Chancellor for Research (VCR), Faculty of Dentistry, Tabriz University of Medical Sciences (TUOMS), Tabriz, Iran.

## Competing Interests

The authors declare that they have no competing interest.

## Ethics Approval

Not applicable.
